# How does the length of cardiopulmonary resuscitation affect brain damage in patients surviving cardiac arrest? A systematic review

**DOI:** 10.1186/s13049-018-0476-3

**Published:** 2018-09-10

**Authors:** Clare Welbourn, Nikolaos Efstathiou

**Affiliations:** 1 College of Medical and Dental Sciences, University of Birmingham, Edgbaston, Birmingham B15 2TT UK; 2 College of Medical and Dental Sciences, Institute of Clinical Sciences, School of Nursing, Medical School, University of Birmingham, Room EF15, Vincent Drive, Birmingham, B15 2TT UK

**Keywords:** Literature review, Systematic review, Cardiopulmonary resuscitation, Advanced life support, Cardiac arrest, Neurological outcome, Time factors, Decision making

## Abstract

**Background:**

Brain injury can occur after cardiac arrest due to the effects of ischaemia and reperfusion. In serious cases this can lead to permanent disability. This risk must be considered when making decisions about terminating resuscitation. There are very specific rules for termination of resuscitation in the prehospital setting however a similar rule for resuscitation in hospital does not exist. The aim of this review was to explore the effects of duration of cardiopulmonary resuscitation on neurological outcome in survivors of both in-hospital and out-of-hospital cardiac arrest achieving return of spontaneous circulation in hospital.

**Methods:**

A systematic review was conducted. Five databases were searched in addition to hand searching the journals Resuscitation and Circulation and reference lists, quality of the selected studies was assessed and a narrative summary of the data presented. Studies reporting relevant outcomes were included if the participants were adults achieving return of spontaneous circulation in the hospital setting. Studies looking at additional interventions such as extracorporeal resuscitation and therapeutic hypothermia were not included. Case studies were excluded. The study period was from January 2010 to March 2016.

**Results:**

Seven cohort studies were included for review. Quality scores ranged from eight to 11 out of 12. Five of the studies found a significant association between shorter duration of resuscitation and favourable neurological outcome.

**Conclusions:**

There is generally a better neurological outcome with a shorter duration of CPR in survivors of cardiac arrest however a cut-off beyond which resuscitation is likely to lead to unfavourable outcome could not be determined and is unlikely to exist. The findings of this review could be considered by clinicians making decisions about terminating resuscitation. This review has highlighted many gaps in the knowledge where future research is needed; a validated and reliable measure of neurological outcome following cardiac arrest, more focused research on the effects of duration on neurological outcome and further research into the factors leading to brain damage in cardiac arrest.

**Electronic supplementary material:**

The online version of this article (10.1186/s13049-018-0476-3) contains supplementary material, which is available to authorized users.

## Background

Cardiac arrest is the cessation of effective contraction of the myocardium leading to sudden loss of consciousness and absence of pulse and respiratory function [1]. In 2010, Berdowski et al. estimated that the global incidence of out of hospital cardiac arrests was 55 arrests per 100,000 person-years [2]. Since the 1960s, cardiopulmonary resuscitation (CPR) has been used to attempt to restart the heart following cardiac arrest [3], however prognosis is poor, with a global average survival rate of 7% [2]. There is currently limited evidence available to clinicians to guide their decisions about continuation of CPR and its possible impact on outcomes.

Cardiac arrest followed by CPR and subsequent return of spontaneous circulation (ROSC) leads to global ischaemia-reperfusion injury. The abrupt cessation of blood flow causes ischaemia and hypoxia. Cell membrane ion transporters, which require oxygen-dependent adenosine triphosphate, stop functioning. Calcium floods into the cells [4], promoting apoptosis, in addition to excitotoxicity, whereby neurotransmitters are over-stimulated leading to neuronal damage [5]. Reperfusion injury occurs when CPR is commenced, bringing about oxidative stress and the formation of free radicals and reactive oxygen species [6]. These are very damaging and react with many macromolecules, including DNA, proteins and lipids. In addition, global reperfusion leads to activation of leukocytes causing an inflammatory stress response, disrupting the blood-brain barrier, causing further damage by fluid leaking into the intracellular space leading to cerebral oedema [6]. CPR partially reverses the ischaemia but it is not as effective as the heart, and cerebral perfusion pressure remains low until ROSC is achieved, at which point further reperfusion damage occurs [7].

Brain damage can seriously impact the lives of survivors of CPR sometimes causing permanent disability. Studies have demonstrated that psychosocial and cognitive impairment are more common in those surviving cardiac arrest with a brain injury; anxiety, depression and post-traumatic stress disorder are increased; and social interaction is reduced [8, 9]. In addition, memory loss is particularly common after cardiac arrest [10]. It is unclear whether ischaemic-reperfusion damage is the direct cause, due to the life-changing impact any critical illness may have. However, it has been demonstrated that the brain regions associated with these problems are more susceptible to anoxic injury [5].

Considering the damaging effects of CPR, the difficult decision about when to terminate resuscitation attempts in those with no ROSC must be made by healthcare professionals. Although extensive research has been conducted in order to create and validate the Termination of Resuscitation rule for the prehospital setting [11], current guidelines for the hospital setting are ambiguous. Both the UK and European Resuscitation Councils suggest considering terminating CPR after 20 min of asystole [12, 13], however this has little empirical support. The American Heart Association (AHA) guidelines simply state that clinical judgement should be used to make the decision [14]. Ultimately potential risks and benefits of carrying on CPR must be weighed up, therefore the possibility of a link between prolonged CPR and brain injury, should influence decisions for CPR termination when there is no ROSC.

Brain injury is always a risk in cardiac arrest patients achieving ROSC, however it is possible that prolonged CPR may cause further damage due to reduced cardiac output during resuscitation. This review seeks to explore whether the risk of brain damage increases with prolonged CPR in the hospital setting. Previous research has looked at various aspects of CPR, though evidence focusing on this area has not been reviewed in a systematic way. The aims of this systematic review were therefore to explore the effects of duration of CPR on neurological outcome in survivors of both in-hospital cardiac arrest (IHCA) and out-of-hospital cardiac arrest (OHCA) achieving ROSC in hospital and to investigate whether there is a maximum duration of CPR to avoid or reduce the risk of unfavourable outcome.

## Methods

In order to conduct a systematic review in a way that minimised bias, the PRISMA statement, which is designed for the reporting of systematic reviews, particularly intervention studies, was followed [15]. A protocol for this systematic review was developed by the author and a reviewer experienced in systematic reviews.

### Search methods

**Fig. 1:**
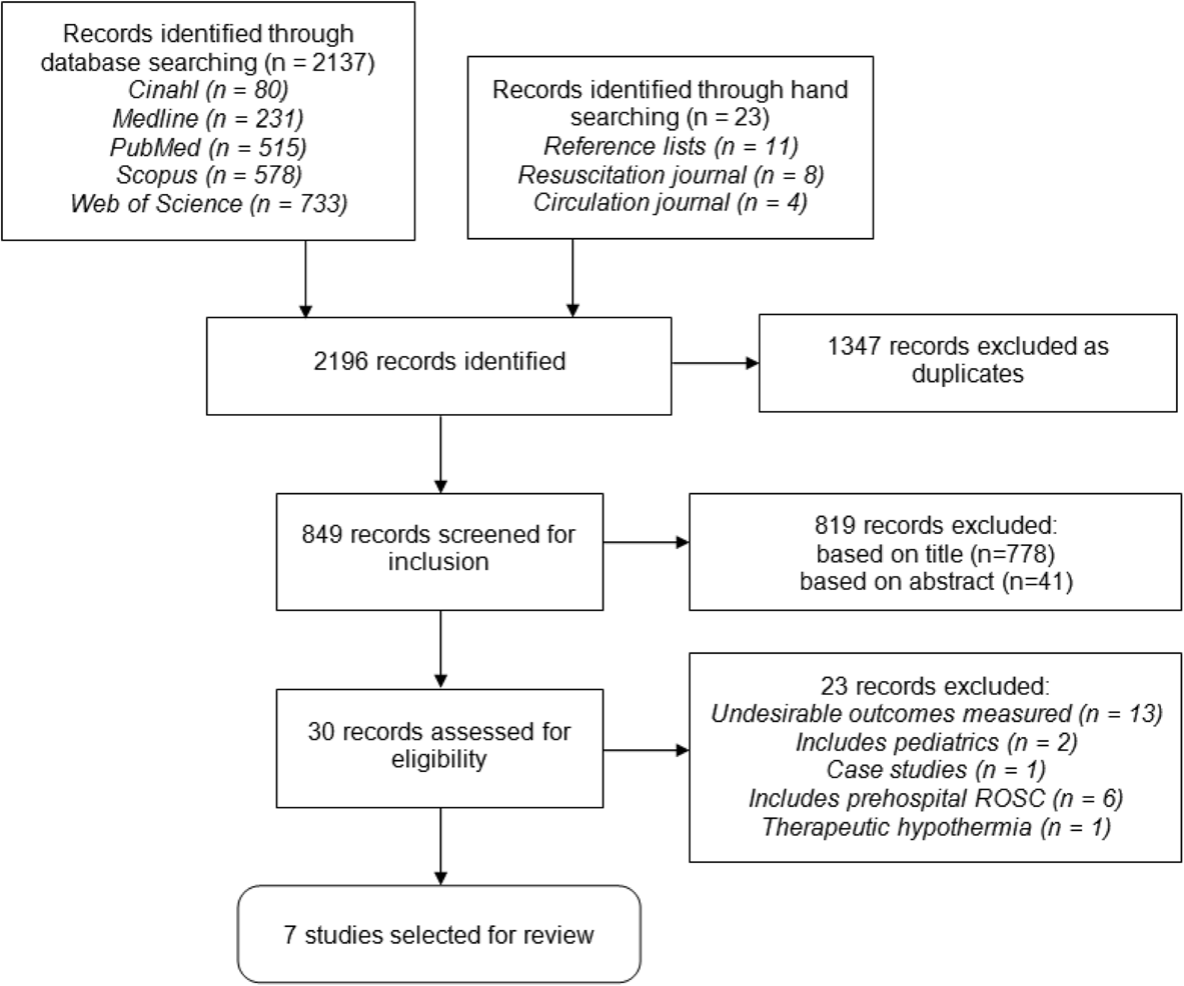
Flowchart of the literature search and selection process

A search of Cinahl, Medline, PubMed, Scopus and Web of Science was conducted in March 2016. Since not all databases used medical subject headings (MeSH), a combination of MeSH terms and keywords were used (Table 1). An example of this is given in Additional file 1: Appendix 1. Creation of the search string was overseen by a subject specific librarian. In addition, reference lists of relevant papers and the journals Resuscitation and Circulation, which are the European Resuscitation Council and AHA’s journals respectively, were hand searched. All retrieved papers were entered on a reference management software (RefWorks), and duplicates were removed. Potentially relevant articles were then screened based on title and then on reading the abstract. If they appeared relevant, the full-text articles were assessed for eligibility (Fig. 1). The literature search period was from January 2010 to March 2016. Inclusion and exclusion criteria for the review are presented in Table 2. Studies reporting CPR on children were not included due to the differences in aetiology and physiology of cardiac arrest [16]. Paediatrics tend to have a much higher survival rate but poorer neurological outcome, therefore this could bias the synthesis of findings of this review [17].

**Table 1:** Search strategy

Database	Keywords			Limits
Cinahl (EbscoHost)	Resuscitation, Cardiopulmonary (MeSH)	duration	neurolog*	Jan 10 – Mar 16
		time	cogniti*	English
			“cerebral performance”	
			“function* outcome”	
Medline (OVID)	Cardiopulmonary resuscitation (MeSH)	duration	neurolog*	Jan 10 – Mar 16
		time	cogniti*	English
			“cerebral performance”	
			“function* outcome”	
PubMed (NCBI)	“Cardiopulmonary resuscitation”	duration	neurolog*	Jan 10 – Mar 16
	“Advanced cardiac life support”	time	cogniti*	English
	“Advanced life support”		“cerebral performance”	
			“function* outcome”	
Web of science	“Cardiopulmonary resuscitation”	duration	neurolog*	Jan 10 – Mar 16
	“Advanced cardiac life support”	time	cogniti*	English
	“Advanced life support”		“cerebral performance”	
			“function* outcome”	
Scopus	“Cardiopulmonary resuscitation”	duration	neurolog*	Jan 10 – Mar 16
	“Advanced cardiac life support”	time	cogniti*	English
	“Advanced life support”		“cerebral performance”	Health sciences
			“function* outcome”	Life sciences

**Table 2:** Eligibility criteria for the selection of papers

	Inclusion	Exclusion
Population	Adults survivors of cardiac arrest receiving resuscitation in hospital	Animal studies; paediatrics
Intervention	CPR duration described as collapse to ROSC	Prehospital ROSC; additional interventions including but not limited to extracorporeal resuscitation, therapeutic hypothermia
Outcomes measured	Neurological outcome of survivors of CPR	Studies in which CPR duration is not compared with neurological outcome
Type of article	Research studies	Case studies
Language	English	Papers not published in English
Date	Published from 01/01/2010	Published prior to 2010

### Data extraction

Information from the included studies was extracted on an Excel data extraction form and consisted of the studies’ characteristics (bibliographic details, aims and objectives of the study, methodology, population and setting) and related findings (Table 3). Data extraction was completed by one author (CW) and overread by the second author (NE). The level of agreement following overreading was high (>85%) and any disagreements were discussed and resolved between the authors.

**Table 3:** Data extraction table for included studies

Author, year of publication	Title	Country of origin	Aims/objectives	Study design	Data collection	n=	Age (yrs) median (IQR) or mean ± SD	Gender (% male)	Inclusion (Incl) Exclusion (Excl)	Type of CA	Location	Outcome measures	Findings
Chan et al., 2012 [20]	A validated prediction tool for initial survivors of in-hospital cardiac arrest	USA (Not stated in study)	to develop a valid and clinically useful risk prediction tool among succesfully resuscitated patients with an IHCA, to estimate favourable neurological survival	Cohort study	National CA database (GWTG-R registry)	42,957	68 (56–78) Mean 66	56	Incl: 2000–2009; adults (≥18 yrs), achieved ROSC Excl: Arrest location ED, OT, post-op, procedural areas; incomplete data	IHCA	USA, multicentre, 551 hospitals	Neurologically favourable survival to discharge measured by CPC score	Duration of CPR is a good predictor of neurological status at discharge
Constant et al., 2014 [21]	Predictors of functional outcome after intraoperative cardiac arrest	France	to identify factors associated with 90-day favourable functional outcomes in adults admitted to the ICU after succesful resuscitation of intra-operative CA	Cohort study	Medical records (Utstein format) interviewing patients, NOK, GP, neurologist	140	60 (46–70)	56.4	Incl: 2000–2013; adults; received anaesthesia, admitted to ICU after succesful resuscitation	IOCA	France, multicentre, 11 hospital ICUs	Functional status at 90 days measured by CPC score	Shorter duration of CPR is associated with a more favourable outcome (CPC 1–2)
Goldberg et al., 2012 [22]	Duration of resuscitation effors and survival after in-hospital cardiac arrest: an observational study	USA	to investigate whether duration of resuscitation attempt varies between hospitals and whether patients at hospitals that attempt resuscitation for longer have higher survival rates than those with shorter durations of resuscitation efforts	Cohort study	National CA database (GWTG-R registry)	64,339 (8724 with CPC score)	69 (57–78)	55.8	Incl: 2000–2008; adults (≥18 yrs); first CA during inpatient stay Excl: ICD; arrest location ED, OT, post-op, procedural areas, rehab areas; <2 mins arrest; incomplete data	IHCA	USA, multicentre, 435 hospitals	Neurological status at discharge measured by CPC score	No significant link between duration of CPR and neurological outcome
Iqbal et al., 2015 [23]	Predictors of survival and favourable outcomes after an out-of-hospital cardiac arrest in patients systematically brought to a dedicated heart attack cener (from the Harefield cardiac arrest study)	UK	to determine the predictors of favourable functional status at discharge and long-term survival in patients experiencing out of hospital CA who are brought to a dedicated heart attack centre	Cohort study	National research database (Utstein-style template) Case notes reviewed for data on functional status	174	65 (56–65)	79.9	Incl: 2011–2013	OHCA	UK, single site, dedicated heart attack centre	Functional status at discharge measured by mRS	Shorter duration of CPR is a powerful predictor of favourable functional outcome at discharge.
Reynolds et al., 2013 [24]	Duration of resuscitation effors and functional outcome after out-of-hospital cardiac arrest when should we change to novel therapies?	USA	to estimate the dynamic probability of survival and functional recovery as a function of resuscitation effort duration to identify when to use novel therapies	Cohort study	Hospital CA database (Utstein-style template)	1014	Mean 65.2	57.7	Incl: 2005–2011; adults (≥18 yrs)	Non-traumatic OHCA	USA, single site, ED	Functional status at discharge measured by mRS	Shorter duration of CPR is independantly associated with survival to d/c with a favourable outcome (mRs of 0–3)
Vancini-Campanharo et al., 2015 [25]	One-year follow-up of neurological status of patients after cardiac arrest seen at the emergency room of a teaching hospital	Brazil	to describe neurological status and associated factors of survivors after CA, upon discharge and at six and twelve month follow ups	Cohort study	Utstein-style recording of consecutive IHCAs Neurological status evaluated with patient, family or guardian	16	not stated	not stated	Incl: 2011–2012; adults (≥17 yrs); resuscitated in ED; survivors to discharge	OHCA	Brazil, single site, city hospital ED	Neurological status at discharge, 1, 6 and 12 months measured by CPC score	No significant link between duration of CPR and neurological outcome
Xue et al., 2013 [26]	Factors influencing outcomes after cardiopulmonary resuscitation in the emergency department	China	to assess the factors influencing outcome of CPR in ED	Cohort study	Hospital registry Utstein-style template	725	46.94 ± 19.05	71.6	Incl: 2005–2011; adults (≥16 yrs) Excl: DNAR; incomplete data	IHCA and OHCA	China, single site, city hospital ED	Neurologically favourable survival to discharge measured by CPC score	CPR ≤15mins had significantly higher percentage of survivors with a neurologically favourable outcome

Abbreviations: *CA* cardiac arrest, *CPC* cerebral performance category, *CPR* cardiopulmonary resuscitation, *d/c* discharge, *ED* emergency department, *GWTG-R* get with the guidelines-resuscitation, *ICD* implantable cardioverter defibrillator, *ICU* intensive care unit, *IHCA* in hospital cardiac arrest, *IOCA* intra-operative cardiac arrest, *mRS* modified Rankin scale, *NOK* next of kin, *OHCA* out of hospital cardiac arrest, *OT* operating theatre, *post-op* post-operative areas, *ROSC* return of spontatneous circulation

### Quality appraisal

The National Institute of Health’s [18] Quality Assessment Tool for Observational Cohort and Cross-Sectional Studies which focuses on key study components such as participants, measurement of variables and control of confounders was selected to appraise the papers, but was adapted to simplify it and increase its relevance to the studies appraised (Table 4). Repeated exposure assessment and follow-up rate were removed as they were not relevant to the study type. The papers were given a score out of 12 based on how many questions were answered favourably. The appraisal was performed by one reviewer (CW) and checked by a second reviewer (NE).

**Table 4:** Quality appraisal tool

Research question	Are the research question and objectives clearly stated?
Recruitment	Are the recruitment methods and study population clearly described?
Baseline measured	Was the outcome of interest measured prior to exposure to gain a baseline for the participants? Was this accounted for when measuring the outcome?
Similar cohorts	Were eligibility criteria applied uniformly across cohorts and all participants recruited from the same or similar population?
Sample size	Is there a sample size justification, power description, or variance and effect estimates provided?
Causation	Was the exposure assessed prior to outcome measurement?
Time-frame	Was there sufficient time-frame to see an effect?
Exposure levels	Did the study examine different levels of the exposure of interest? (i.e. multiple categories of exposure or exposure measured as a continuous variable)?
Exposure measurement	Were the exposures (independent variables) measured in a way that minimised bias? Were they clearly defined, valid, reliable, and implemented consistently across all study participants?
Outcome measurement	Were the outcomes (dependent variables) measured in a way that minimised bias? Were they clearly defined, valid, reliable, and implemented consistently across all study participants?
Blinding	Were assessors blinded to exposure? (Where researchers are using data already collected, this would be yes)
Confounders	Were key potential confounding variables identified and controlled for in statistical analysis? (i.e. were regression models used?)

### Analysis

Quantitative content analysis of the studies’ findings was undertaken as a meta-analysis was inappropriate due to the lack of homogeneity between the studies [19]. In addition to the primary outcome of interest, which was the duration of CPR and associated neurological outcome, secondary outcomes including age, gender, initial rhythm and location of arrest were considered in the analysis.

## Results

### Search outcomes

Following a systematic literature search in five databases, 2137 studies were found in total, with an additional 23 studies found from hand searching. After removing duplicates, 849 studies remained. These were screened for relevance based on title and abstract. Based on title, 71 articles appeared relevant and a further 30 were selected based on their abstract. Twenty-three of these were discarded for not meeting the eligibility criteria for this review. The most frequent reasons for exclusion were that outcomes were not relevant to this review (Fig. 1). A total of seven studies were included in the review and were appraised for quality [20–26].

All of the papers were cohort studies and generally used data collected routinely through national or hospital registries. Sample sizes ranged from 16 to 64,339. Out of the six studies which reported participants’ demographics, five had an average age of participants of over 60 years, with the sixth study’s participants considerably younger at 47 years. All had a greater number of males than females. Three studies reported findings from OHCA, three from IHCA and one study included both OHCA and IHCA. Four of the studies were conducted at a single site and three were multicentre, with the majority conducted in Europe and the US. Five of the studies used Cerebral Performance Category (CPC) and two used the modified Rankin Scale (mRS) to measure neurological outcome. Five studies measured the outcome at discharge, one measured outcome at discharge with a follow-up at one, six and 12 months and one measured 90 day outcome (Table 3).

### Quality appraisal findings

The papers were given quality scores ranging from eight to 11 out of 12 (Table 5), using the appraisal process described. All studies had clear objectives. Appropriate approach and design were always used, but not always clearly stated. The quality of selection of participants was mixed. Five studies clearly demonstrated their recruitment and sampling methods with eligibility criteria and had a clear description of the cohort in terms of age and sex [20–24]. Other demographics such as ethnicity were not usually included. In five of the studies it was unclear whether any neurological deficit prior to cardiac arrest was accounted for [21–24, 26]. However this may have had little impact as Chan et al. [20] found that when restricting the cohort to include only those with a favourable neurological status it made little to no difference to the overall results. All of the studies recruited participants from similar cohorts. None of the studies provided justification for the choice of sample size however many of the studies had a large sample, therefore this does not necessarily reflect low quality. The study by Vancini-Campanharo [25] had a small sample size of *n* = 16, and received a relatively low appraisal score, therefore it was not included in the comparative analysis due to high risk of bias.

**Table 5:** Quality appraisal outcomes

Author, year	Research question	Recruitment	Baseline measured	Similar cohorts	Sample size	Causation	Time-frame	Exposure levels	Exposure measurement	Outcome measurement	Blinding	Confounders	Score
Chan et al., 2012 [20]	y	y	y	y	n	y	y	y	y	y	y	y	11
Constant et al., 2014 [21]	y	y	unclear	y	n	y	y	y	y	y	unclear	y	9
Goldberger et al., 2012 [22]	y	y	unclear	y	n	y	y	y	y	y	y	y	10
Iqbal et al., 2015 [23]	y	y	unclear	y	n	y	y	y	y	y	y	y	10
Reynolds et al., 2013 [24]	y	y	unclear	y	n	y	y	y	y	y	y	y	10
Vancini-Campanharo et al., 2015 [25]	y	n	y	y	n	y	y	y	y	y	unclear	n	8
Xue et al., 2013 [26]	y	n	unclear	y	n	y	y	n	y	y	y	y	8

Each of the studies reported a rigorous approach to measurement of the variables. In all studies exposure was assessed prior to outcome, which provides stronger evidence that the exposure caused the outcome. All studies had sufficient time-frame. All except one study [26] used different levels of exposure, either by using several time-categories or time as a continuous variable. All studies used Utstein-style reporting to minimise the risk of bias when measuring both CPR duration and neurological outcome. Five were blinded to the outcome [20, 22–24, 26], as the data was not collected by the researchers; for the other two studies this is unclear [21, 25]. All studies had clear statistical methods and six of the studies controlled for confounding variables in their analysis [20–24, 26].

### Data synthesis findings

Five studies found a significant link between shorter duration and favourable neurological outcome (CPC 1–2 or mRS 0–3) [20, 21, 23, 24, 26]. One study found no significant link between rate of favourable outcome and CPR duration but did find a worse outcome in those with a longer duration when looking at mean and median scores. This was one of the highest quality studies and had a very large, representative study population.

There was no obvious difference in neurological outcomes between the studies looking at OHCA and IHCA. Of the three studies looking solely at IHCA, two found a significant link [20, 21] and the other did not. The two studies including only OHCA both found a significant link between duration and neurological outcome. Three of the studies looked at the average duration of favourable and unfavourable outcomes [21, 23, 24]. One of these studied IHCA and had an average of 6 minutes of CPR for a good outcome compared to 15 minutes for a poor outcome. Similarly the two OHCA studies had an average duration of four and 6.2 min for good outcome and 16 minutes in both studies for a poor outcome. One study including both IHCA and OCHA found that patients who had an OHCA had a significantly worse outcome than IHCA.

None of the studies looked at characteristics of those with and without neurological deficit at different durations, however many of the studies did look at the association of various variables with neurological outcome. There was mixed evidence on the effect of age. Of the five studies which looked at age, three found that older age is significantly linked with poorer outcome [20, 23, 24] and two found no significant link [21, 26]. Four studies also looked at gender and found no significant link to outcome [21, 23, 24, 26]. The most notable other factor which was investigated in all studies was the relationship between neurological outcome and shockable (ventricular fibrillation or pulseless ventricular tachycardia) or non-shockable (asystole or pulseless electrical activity) rhythm. Shockable rhythm was almost always associated with a significantly better neurological outcome. The only exception to this was in one study which found this only to be the case in OHCA. Only one of the highest quality studies looked at the neurological outcomes of patients with shockable and non-shockable rhythms at different durations and found that duration had a greater impact on shockable than non-shockable rhythms.

## Discussion

This systematic review found seven studies of varying quality reporting on duration of CPR and neurological outcome. Generally, neurological outcomes were better in patients who achieved ROSC after a shorter time, however this review has revealed no definitive maximum duration, beyond which CPR may be futile. Due to the heterogeneity of data interpretation, analyses and reported outcomes, it was not possible to determine a time beyond which resuscitation would be unlikely to yield a favourable outcome. There was insufficient evidence to determine a meaningful difference between OHCA and IHCA. There was considerable variation in findings when looking at age as a factor in neurological outcome, though findings that gender is irrelevant were conclusive. Shockable rhythm was a significant predictor of favourable outcome.

Most of the studies confirmed that more favourable outcomes were associated with shorter duration of CPR. In part, this echoes the systematic review by Moulaert et al. [9] which investigated duration as a confounding variable to cognitive impairment following OHCA. Of the two studies which identified confounding variables, both demonstrated an association between time to ROSC and cognitive outcome. However, in contrast to our findings, four studies in Moulaert et al.’s review found no confounding variables. By using number of doses of adrenaline and number of shocks as proxy markers, Kaye [27] associated better outcomes with shorter durations, however caution must be applied as the methodology was unclear and of poor quality. Similar findings have been reported in the prehospital setting; for example both Abe et al. [28] and Grunau et al. [29] found that favourable neurological outcome is more likely with a shorter time to ROSC. There were some differences between those experiencing ROSC in the prehospital and the hospital setting. Abe et al. [28] and Matsuyama et al. [30] found when looking at patients with good outcomes, CPR duration was shorter in those with prehospital ROSC. It would be interesting to explore whether this is influenced by the Termination of Resuscitation rule for the prehospital setting.

Xue et al. [26] found that there was a significantly better neurological outcome in those who had an IHCA compared with those with OHCA. They also reported that arrests witnessed by medical staff had a significantly better neurological outcome. Both of these findings are consistent with greater likelihood that time between arrest and commencement of CPR was relatively short. Iqbal et al. [23] found that bystander CPR also had a significant impact on neurological outcome. It may be that the increased period of hypoxia whilst no CPR is being carried out leads to brain damage further exacerbated by reperfusion injury. However Storm et al. [31] when investigating the effect of cerebral oxygenation during CPR found that a low value at the beginning of treatment on arrival of emergency services was not a good predictor of ROSC or neurological outcome. In contrast, Parnia et al. [32] found that in IHCA, cerebral oxygenation values were a significant predictor of a neurologically favourable survival.

Despite generally finding a significant correlation between duration and neurological outcome, the incidence of complete recovery after prolonged CPR is high. For example Goldberger et al. [22] found that 73.8% of people receiving CPR for more than 30 min survived neurologically intact. Case studies, which often report remarkable outcomes, were excluded from this review due to the risk of publication bias however their findings can be interesting and useful. In a review of all published cases of patients who underwent prolonged CPR of greater than 20 minutes, 78% recovered with a favourable neurological outcome [33]. The median duration of resuscitation in the reviewed cases by Youness [33] was 75 min with a range of 20–330 min. In these cases it appears that duration had little impact on outcome. It is fair to conclude it would be unethical to specify a maximum duration after which CPR should be terminated.

The study by Goldberger et al. [22] found no significant link between the rate of favourable neurological outcome and duration of resuscitation. This was a high-quality study, with a very large sample size and has been widely referenced, including by the Resuscitation Council (UK) [34]. However they did find that mean and median CPC scores were higher in those who had a shorter duration. It is possible to reach very different conclusions depending on whether selecting mean CPC score (*p* = 0.0001) or proportion of people with favourable outcome (*p* = 0.131) when interpreting the data. Despite the similarities in data collection between the studies, there was considerable variation in data interpretation and presentation of results. Arguably, it is potentially more meaningful to focus on the proportion of people with a good or bad outcome than average CPC score because of the discrete nature of the CPC scale. Goldberger et al.’s [22] results are consistent with two separate population groups – one with a good prospect of recovery, in which duration of CPR had little effect, and a larger second group with poorer prospects of recovery, and amongst whom damage was more likely to increase with time of CPR. This hypothesis could explain their apparently conflicting results in which average CPC score correlates with CPR time, but percentage of good outcomes does not.

If this interpretation is correct, it has important implications. If the patient is likely to have a good outcome then prolonged CPR is justifiable, whereas in those cases where the arrest is likely to have a poor outcome this may worsen with prolonged CPR. It is therefore important to better understand other arrest factors which have an impact on outcome. In Youness et al.’s [33] study of prolonged CPR, the participants were generally young, with no co-morbidities and had cardiac arrest with reversible causes, however these findings are not discussed in depth and further research is needed.

Significance between shockable rhythm and favourable outcome was identified across all studies in this review. Three large (*n* = 30,716, 64,339 and 91,658), good quality studies, exploring CPR duration, found an association between shockable rhythm and shorter duration of resuscitation as a predictor of favourable neurological outcome [22, 35, 36]. This may be an indicator of the importance of cause of arrest in likelihood of survival with a good outcome. However, little research has been done to investigate the link between initial rhythm and neurological outcome with prolonged CPR.

Only one of the papers in this review considered institutional duration of CPR. Goldberger et al. [22] found a higher overall survival rate in those hospitals which had a longer average duration of CPR, but found no difference between hospitals when looking at favourable neurological outcome to discharge. Cha et al. [36] similarly found a higher survival rate with longer institutional duration of CPR. This implies that if CPR were attempted for longer there may be a higher survival rate, which contradicts the majority of findings from this review. However Cha et al. did not report these findings in relation to neurological outcome of survivors. Hospitals which resuscitate for longer may give better quality resuscitation and more aggressive treatments which may lead to increased survival [36]. This is an interesting area for future research.

All the studies in this review adopted the Utstein-style for data collection. This is the internationally standardised format for reporting cardiac arrest data for both OHCA and IHCA [37], however, there is limited research demonstrating its validity and reliability. According to Utstein-style reporting, neurological outcome following cardiac arrest should be recorded using either CPC or mRS [37]. These outcome measures are used in all the studies reviewed. There is no evidence to justify the assumption that this should improve the validity of the studies’ findings. Studies have found a lack of validity and reliability of CPC and mRS due to significant variability between the two; limited ability to differentiate between levels of outcome; and lack of focus on any specific aspect of functioning [38–40]. This may have affected the quality of our findings which would have been more reliable had there been a standardised measure for neurological outcome implemented across clinical practice.

Since the searches were conducted there has been additional research published which would have met the inclusion criteria for this study. Four studies, all set in the emergency department were found; one focused on IHCA [41] two on OHCA [42, 43] and one studied both IHCA and OHCA [44]. All four studies found that increased duration of CPR led to a significantly poorer neurological outcome, which was measured by CPC in three of the studies [41–43] and by ability to follow commands in the fourth [44]. The inclusion of these more recent studies would not have changed the conclusions of this review.

This review has identified some interesting findings that require further investigation. It is unclear why some survivors of prolonged resuscitation had complete neurological recovery whilst others did not and further research focusing on duration of CPR, neurological outcome and the factors that affect these may help to answer this.

### Strengths and limitations

To find all the literature on this topic, thorough, systematic searches were conducted. The risk of missing potentially relevant articles when searching was minimised by searching five different databases and hand searching relevant journals and reference lists. Creating a search strategy and selection of papers was only carried out by one reviewer which is a limitation of this study, however this was overseen by a subject specific librarian and approved by a second reviewer.

The inclusion and exclusion criteria may be a further limitation for this study. The search was limited to papers in English which could introduce language bias. Due to frequent changes in CPR guidelines and ever-improving outcomes, the search was limited to studies published after 2010 in order to keep a relatively narrow time-frame in which practices could be assumed to remain fairly consistent. Wang et al. [45] found a higher probability of favourable neurological outcome with CPR conducted after 2010 due to the vast changes in guidelines that year, however only two of the studies were based entirely on data collected since 2010 with some including results reported in 2000. There is clearly a risk of variation associated with changes in practice. Therapies supplementary to advanced life support such as extracorporeal resuscitation or therapeutic hypothermia were excluded from the review. These may have an important effect on outcomes, but would have led to a much more complex review with difficulty isolating the findings. Excluding this potentially large volume of literature means that caution should be used in extrapolating the findings to this population. Excluding patients who achieved prehospital ROSC may have introduced bias, however papers investigating prehospital ROSC report similar findings to those studies included for review [28–30].

The similarity in the design and methods of the studies allowed comparisons to be drawn using the same appraisal tool across the studies, maintaining objectivity and minimising bias. All parameters in the chosen appraisal tool were equally weighted despite the possibility of some having greater influence in the overall quality than others. The appraisal tool highlighted the main areas in which bias could have been introduced but did not discriminate between large and small flaws. An alternative may have been to use a scale however this may be more subjective.

The included studies relied on retrospective collection of registry data. There is potential for errors in data collection, variation of recording methods between hospitals or misinterpretation of data [22, 35]. It would be highly unethical to conduct experimental studies in this area of research. As with any systematic review, there is a risk of publication bias as many papers will only report significant findings. With only a small number of relevant studies, it proved impossible to restrict studies to only those of the highest quality. The limited similarity between studies prevented conducting a meta-analysis.

Overall this literature review included a number of steps to maintain quality. Bias was minimised by following the PRISMA procedure with minimal deviation. Reporting of methods was transparent throughout to increase replicability. Consistency of findings amongst the majority of the studies increases confidence in the findings of this review. The findings are generalisable to the study population, as both IHCA and OHCA in most hospital settings, all arrest types and a wide variety of hospitals and locations were included. The review sought only to study the adult population and therefore the findings cannot be applied to paediatrics.

## Conclusions

Current guidelines on terminating in-hospital resuscitation are discussed very briefly, leaving healthcare professionals to use clinical judgement as the main factor when making these decisions. It is hoped that in the future, enough conclusive evidence from quality research will lead to provision of clearer guidance on terminating resuscitation in the hospital setting. This systematic review sought to find out whether duration of CPR has an impact on neurological outcome of survivors of cardiac arrest. Seven studies were included for review. These were appraised for quality and were mostly of a high standard.

### Key findings

- There is generally a better neurological outcome with a shorter duration of CPR in survivors of cardiac arrest, however a cut-off beyond which resuscitation is likely to lead to unfavourable outcome was not possible to determine and is unlikely to exist, as many people survive prolonged cardiac arrest with minimal consequences.
- There is not enough evidence to create a definitive rule for termination of CPR in the hospital setting. Clinicians should continue to take into account that in many cases the chance of neurologically favourable survival decreases the longer CPR is continued, however this alone is not enough to make the decision to terminate efforts.
- There is a need for a validated and reliable measure of neurological outcome following cardiac arrest.
- Future research is required in several areas in order for more specific guidelines around the duration of resuscitation attempts to be created.

## Additional file

Additional file 1: Appendix 1. Search carried out in Cinahl (EbscoHost) (DOCX 19 kb)

## Abbreviations

AHA: American Heart Association
CPC: Cerebral performance category
CPR: Cardiopulmonary resuscitation
IHCA: In-hospital cardiac arrest
MeSH: Medical subject headings
mRS: Modified Rankin Scale
OHCA: Out-of-hospital cardiac arrest
ROSC: Return of spontaneous circulation

## References

[1] Martin E (2008). McFerran T.

[2] Berdowski J, Berg RA, Tijssen JGP, et al. Global incidences of out-of-hospital cardiac arrest and survival rates: systematic review of 67 prospective studies*.* Resuscitation 2010;81:1479-87 doi:10.1016/j.resuscitation.2010.08.006. 10.1016/j.resuscitation.2010.08.006 20828914

[3] Safar P, Elam JO, Jude JR (1963). Resuscitative principles for sudden cardiopulmonary collapse. Chest.

[4] Grubb NR (2001). Managing out-of-hospital cardiac arrest survivors: 1. Neurological perspective. Heart.

[5] O'Neil BJ, Koehler RC, Neumar RW, Paradis NA, Halperin HR, Kern KB (2007). Global brain ischaemia and reperfusion. Cardiac arrest: the science and practice of resuscitation medicine.

[6] Hamann K, Beiser T, Vanden Hock TL, Paradis NA, Halperin HR, Kern KB (2007). Global cellular ischaemia/reperfusion during cardiac arrest: critical stress responses and the postresuscitation syndrome. Cardiac arrest: the science and practice of resuscitation medicine.

[7] Nolan JP, Neumar RW, Adrie C, et al. Post-cardiac arrest syndrome: Epidemiology, pathophysiology, treatment, and prognostication: A Scientific Statement from the International Liaison Committee on Resuscitation; the American Heart Association Emergency Cardiovascular Care Committee; the Council on Cardiovascular Surgery and Anesthesia; the Council on Cardiopulmonary, Perioperative, and Critical Care; the Council on Clinical Cardiology; the Council on Stroke*.* Resuscitation 2008;79:350–79 doi:10.1016/j.resuscitation.2008.09.017. 10.1016/j.resuscitation.2008.09.017 18963350

[8] Wilson M, Staniforth A, Till R, et al. The psychosocial outcomes of anoxic brain injury following cardiac arrest*.* Resuscitation 2014;85:795–800 doi:10.1016/j.resuscitation.2014.02.008. 10.1016/j.resuscitation.2014.02.008 24560827

[9] Moulaert VRMP, Wachelder EM, Verbunt JA (2010). Determinants of quality of life in survivors of cardiac arrest. J Rehabil Med.

[10] van Alem AP, de Vos R, Schmand B, et al. Cognitive impairment in survivors of out-of-hospital cardiac arrest*.* Am Heart J 2004;148:416–21 doi:10.1016/j.ahj.2004.01.031. 10.1016/j.ahj.2004.01.031 15389227

[11] Goto Y, Maeda T, Goto YN (2013). Termination-of-resuscitation rule for emergency department physicians treating out-of-hospital cardiac arrest patients: an observational cohort study. Crit Care.

[12] Soar J, Nolan JP, Böttiger BW, et al. European Resuscitation Council Guidelines for Resuscitation 2015: Section 3. Adult advanced life support*.* Resuscitation 2015;95:100–47 doi:10.1016/j.resuscitation.2015.07.016. 10.1016/j.resuscitation.2015.07.016 26477701

[13] Adult advanced Life Support. 2015. Available at: https://www.resus.org.uk/resuscitation-guidelines/adult-advanced-life-support/. Accessed 29 Jan 2016.

[14] Mancini ME, Diekema DS, Hoadley TA (2015). Part 3: ethical issues: 2015 American Heart Association guidelines update for cardiopulmonary resuscitation and emergency cardiovascular care. Circulation.

[15] Moher D, Liberati A, Tetzlaff J, et al. Preferred reporting items for systematic reviews and meta-analyses: The PRISMA statement*.* Int. J. Surg. 2010;8:336–41 doi:10.1016/j.ijsu.2010.02.007. 10.1016/j.ijsu.2010.02.007 20171303

[16] Donoghue AJ, Nadkarni V, Berg RA, et al. Out-of-Hospital Pediatric Cardiac Arrest: An Epidemiologic Review and Assessment of Current Knowledge*.* Ann Emerg Med 2005;46:512–22 doi:10.1016/j.annemergmed.2005.05.028. 10.1016/j.annemergmed.2005.05.028 16308066

[17] Zwingmann J, Mehlhorn AT, Hammer T (2012). Survival and neurologic outcome after traumatic out-of-hospital cardiopulmonary arrest in a pediatric and adult population: a systematic review. Crit Care.

[18] National Institutes of Health. Quality assessment tool for observational cohort and cross-sectional studies. 2014. Available at: http://www.nhlbi.nih.gov/health-pro/guidelines/in-develop/cardiovascular-risk-reduction/tools/cohort. Accessed 18 Feb 2016.

[19] Boland A, Cherry MG, Dickson R (2013). Doing a systematic review: a student's guide.

[20] Chan PS, Spertus JA, Krumholz HM (2012). A validated prediction tool for initial survivors of in-hospital cardiac arrest. Arch Intern Med.

[21] Constant A, Montlahuc C, Grimaldi D (2014). Predictors of functional outcome after intraoperative cardiac arrest. Anesthesiology.

[22] Goldberger ZD, Chan PS, Berg RA, et al. Duration of resuscitation efforts and survival after in-hospital cardiac arrest: an observational study*. Lancet* 2012;380 North American Edition:1473,1481 9p 10.1016/S0140-6736(12)60862-9. 10.1016/S0140-6736(12)60862-9 PMC3535188 22958912

[23] Iqbal MB, Al-Hussaini A, Rosser G (2015). Predictors of survival and favourable functional outcomes after an out-of-hospital cardiac arrest in patients systematically brought to a dedicated heart attack center (from the Harefield cardiac arrest study). Am J Cardiol.

[24] Reynolds JC, Frisch A, Rittenberger JC (2013). Duration of resuscitation efforts and functional outcome after out-of-hospital cardiac arrest when should we change to novel therapies?. Circulation.

[25] Vancini-Campanharo CR, Vancini RL, de Lira CA (2015). One-year follow-up of neurological status of patients after cardiac arrest seen at the emergency room of a teaching hospital. Einstein (Sao Paulo).

[26] Xue JK, Leng QY, Gao YZ (2013). Factors influencing outcomes after cardiopulmonary resuscitation in emergency department. World J Emerg Med.

[27] Kaye P (2005). Early prediction of individual outcome following cardiopulmonary resuscitation: systematic review. Emerg Med J.

[28] Abe T, Tokuda Y, Cook EF (2011). Time-based partitioning model for predicting neurologically favorable outcome among adults with witnessed bystander out-of-hospital CPA. PLoS One.

[29] Grunau B, Reynolds JC, Scheuermeyer FX, et al. Comparing the prognosis of those with initial shockable and non-shockable rhythms with increasing durations of CPR: Informing minimum durations of resuscitation*. Resuscitation* 2016;101:50–6 doi:S0300–9572(16)00047–2. 10.1016/j.resuscitation.2016.01.021 26851705

[30] Matsuyama T, Kitamura T, Kiyohara K, Nishiyama C, Nishiuchi T, Hayashi Y, Kawamura T, Ohta B, Iwami T (2017). Impact of cardiopulmonary resuscitation duration on neurologically favourable outcome after out-of-hospital cardiac arrest: a population-based study in Japan. Resuscitation.

[31] Storm C, Wutzler A, Trenkmann L, Krannich A, von Rheinbarben S, Luckenbach F, Nee J, Otto N, Schroeder T, Leithner C: Good neurological outcome despite very low regional cerebral oxygen saturation during resuscitation—a prospective preclinical trial in 29 patients. Scandinavian Journal of Trauma, Resuscitation and Emergency Medicine 2016, 24. 10.1186/s13049-016-0234-3 PMC4822235 27048406

[32] Parnia S, Yang J, Nguyen R, Ahn A, Zhu J, Inigo-Santiago L, Nasir A, Golder K, Ravishankar S, Bartlett P, Xu J, Pogson D, Cooke S, Walker C, Spearpoint K, Kitson D, Melody T, Chilwan M, Schoenfeld E, Richman P, Mills B, Wichtendahl N, Nolan J, Singer A, Brett S, Perkins G, Deakin C (2016). Cerebral Oximetry during cardiac arrest. Crit Care Med.

[33] Youness H, Al Halabi T, Hussein H, et al. Review and Outcome of Prolonged Cardiopulmonary Resuscitation. Critical Care Research and Practice 2016. 10.1155/2016/7384649 PMC4738728 26885387

[34] Resuscitation Council (UK). Comments on the duration of CPR following the publication of 'Duration of resuscitation efforts and survival after in-hospital cardiac arrest: an observational study' Goldberger ZD et al. Lancet. 2012. Available at: https://www.resus.org.uk/research/other-research/duration-of-resuscitation-efforts-and-survival-after-in-hospital/. Accessed 15 Feb 2016.

[35] Khan AM, Kirkpatrick JN, Yang L (2014). Age, sex, and hospital factors are associated with the duration of cardiopulmonary resuscitation in hospitalized patients who do not experience sustained return of spontaneous circulation. J Am Heart Assoc.

[36] Cha WC, Lee EJ, Hwang S. The duration of cardiopulmonary resuscitation in emergency departments after out-of-hospital cardiac arrest is associated with the outcome: A nationwide observational study*.* Resuscitation 2015;96:323–7 doi:10.1016/j.resuscitation.2015.05.005. 10.1016/j.resuscitation.2015.05.005 25986336

[37] Perkins GD, Jacobs IG, Nadkarni VM (2015). Cardiac Arrest and Cardiopulmonary Resuscitation Outcome Reports: Update of the Utstein Resuscitation Registry Templates for Out-of-Hospital Cardiac Arrest: A Statement for Healthcare Professionals From a Task Force of the International Liaison Committee on Resuscitation (American Heart Association, European Resuscitation Council, Australian and New Zealand Council on Resuscitation, Heart and Stroke Foundation of Canada, InterAmerican Heart Foundation, Resuscitation Council of Southern Africa, Resuscitation Council of Asia); and the American Heart Association Emergency Cardiovascular Care Committee and the Council on Cardiopulmonary, Critical Care, Perioperative and Resuscitation. Circulation.

[38] Mak M, Moulaert VRM, Pijls RW, et al. Measuring outcome after cardiac arrest: construct validity of Cerebral Performance Category*.* Resuscitation 2016;100:6–10 doi:http://dx.doi.org/10.1016/j.resuscitation.2015.12.005. 10.1016/j.resuscitation.2015.12.005 26744101

[39] Rittenberger JC, Raina K, Holm MB, et al. Association between Cerebral Performance Category, Modified Rankin Scale, and discharge disposition after cardiac arrest*.* Resuscitation 2011;82:1036–1040 doi:http://dx.doi.org/10.1016/j.resuscitation.2011.03.034. 10.1016/j.resuscitation.2011.03.034 PMC3138855 21524837

[40] Raina KD, Callaway C, Rittenberger JC, et al. Neurological and functional status following cardiac arrest: Method and tool utility*.* Resuscitation 2008;79:249–56 doi:http://dx.doi.org/10.1016/j.resuscitation.2008.06.005. 10.1016/j.resuscitation.2008.06.005 PMC2600809 18692288

[41] Tan S, Leong B (2018). Cardiac arrests within the emergency department: an Utstein style report, causation and survival factors. Eur J Emerg Med.

[42] Kim J, Kim K, Callaway C, Doh K, Choi J, Park J, Jo Y, Lee J (2017). Dynamic prediction of patient outcomes during ongoing cardiopulmonary resuscitation. Resuscitation.

[43] Balcı K, Balcı M, Şen F, Akboğa M, Kalender E, Yılmaz S: Predictors of neurologically Favourable survival among patients with out-of-hospital cardiac arrest: a tertiary referral hospital experience. Turk Kardiyoloji Dernegi Arsivi-Archives of the Turkish Society of Cardiology 2017. 10.5543/tkda.2017.68480 28429693

[44] Chokengarmwong N, Ortiz L, Raja A, Goldstein J, Huang F, Yeh D (2016). Outcome of patients receiving CPR in the ED of an urban academic hospital. Am J Emerg Med.

[45] Wang C, Huang C, Chang W, Tsai M, Yu P, Wu Y, Chen W (2017). Outcomes of adults with in-hospital cardiac arrest after implementation of the 2010 resuscitation guidelines. Int J Cardiol..

