# Supplementary material for: How does the length of cardiopulmonary resuscitation affect brain damage in patients surviving cardiac arrest? A systematic review
Source: Scand J Trauma Resusc Emerg Med. 2018 Sep 10;26:77. doi: 10.1186/s13049-018-0476-3 (PMC6131783; doi:10.1186/s13049-018-0476-3)
Supplement: Supplementary file 1 — Appendix 1. Search carried out in Cinahl (EbscoHost) (DOCX 19 kb) [file 13049_2018_476_MOESM1_ESM.docx]

**Appendix 1: Search carried out in Cinahl (EbscoHost)**

| **#** | **Search Terms** | **Results** |
| --- | --- | --- |
| 1 | (MH "Resuscitation, Cardiopulmonary") | 6,137 |
| 2 | duration | 49,306 |
| 3 | time | 300,291 |
| 4 | 2 OR 3 | 331,360 |
| 5 | neurolog* | 34,487 |
| 6 | cogniti* | 71,621 |
| 7 | "cerebral performance" | 126 |
| 8 | "function* outcome" | 3,456 |
| 9 | 5 OR 6 OR 7 OR 8 | 105,644 |
| 10 | 1 AND 4 AND 9 | 157 |
| 11 | Limit 10 to - Published Date: 20100101-; English Language | 80 |
